# Supplementary material for: Frequency of Circulating Tumor Cells (CTC) in Patients with Brain Metastases: Implications as a Risk Assessment Marker in Oligo-Metastatic Disease
Source: Cancers (Basel). 2018 Dec 19;10(12):527. doi: 10.3390/cancers10120527 (PMC6315958; doi:10.3390/cancers10120527)
Supplement: Supplementary file 1 [file cancers-10-00527-s001.pdf]

Supplementary Materials: Frequency of Circulating Tumor Cells (CTC) in Patients with Brain Metastases: Implications as a Risk Assessment Marker in Oligo-Metastatic Disease

Annkathrin Hanssen, Carlotta Riebensahm, Malte Mohme, Simon A. Joosse, Janna-Lisa Velthaus, Lars Arne Berger, Christian Bernreuther, Markus Glatzel, Sonja Loges, Katrin Lamszus, Manfred Westphal, Sabine Riethdorf, Klaus Pantel and Harriet Wikman

Table S1. Multivariate analysis.

**All cases**	**Cut off 2 ≥ CTC**	**HR**	***p*-value**	**95% CI**
	CellSearch	2.711	**0.024**	1.137–6.465
	brain	3.295	**0.002**	1.530–7.096
	adren	1.271	0.612	0.503–3.213
	bone	1.624	0.260	0.699–3.776
	lung	1.033	0.925	0.527–2.026
**All cases**	**Cut off 5 ≥ CTC**	**HR**	***p*-value**	**95% CI**
	CellSearch	2.846	**0.030**	1.104–7.339
	brain	3.127	**0.003**	1.476–6.622
	adren	1.501	0.361	0.628–3.585
	bone	1.413	0.449	0.578–3.451
	lung	1.120	0.738	0.576–2.177
**Brain Metastases**	**Cut off 2 ≥ CTC**	**HR**	***p*-value**	**95% CI**
	CellSearch	4.694	**0.004**	1.650–13.354
	adren	5.797	**0.001**	2.018–16.651
	bone	2.904	0.070	0.918–9.190
	lung	0.503	0.177	0.186–1.365
**Brain Metastases**	**Cut off 5 ≥ CTC**	**HR**	***p*-value**	**95%CI**
	CellSearch	4.963	**0.003**	1.752–14.061
	adren	5.750	**0.001**	1.999–16.540
	bone	2.848	0.076	0.896–9.054
	lung	0.499	0.173	0.184–1.356
**Lung Metastases**	**Cut off 2 ≥ CTC**	**HR**	***p*-value**	**95%CI**
	CellSearch	4.547	0.069	0.890–23.220
	Adren	2.093	0.799	0.342-12.820
	Bone	4.650	0.181	0.489–44.181
	Brain	2.677	0.250	0.499–14.362
**Lung Metastases**	**Cut off 5 ≥ CTC**	**HR**	***p*-value**	**95% CI**
	CellSearch	36.790	**0.009**	2.455–551.35
	adren	4.417	**0.049**	1.004–19.44
	bone	2.803	0.415	0.236–33.37
	brain	2.105	0.356	0.433–10.24

© 2018 by the authors. Licensee MDPI, Basel, Switzerland. This article is an open access article distributed under the terms and conditions of the Creative Commons Attribution (CC BY) license (http://creativecommons.org/licenses/by/4.0/).
